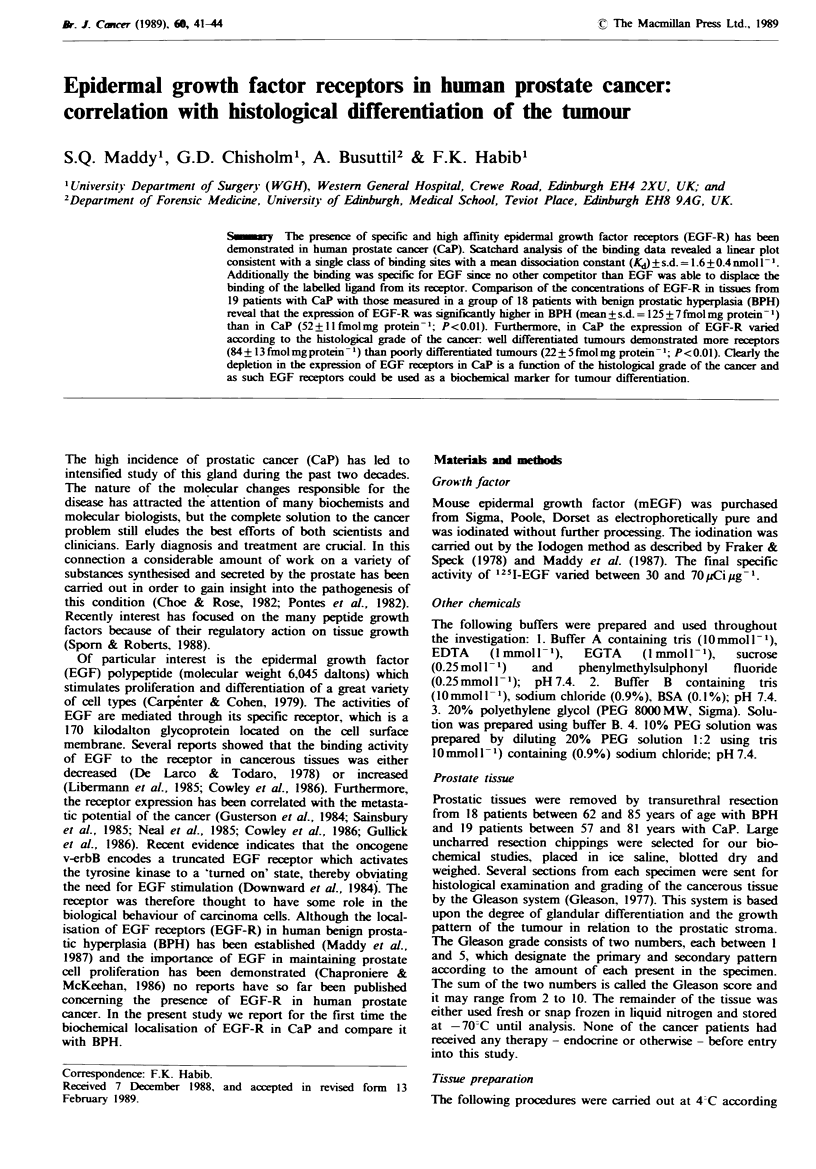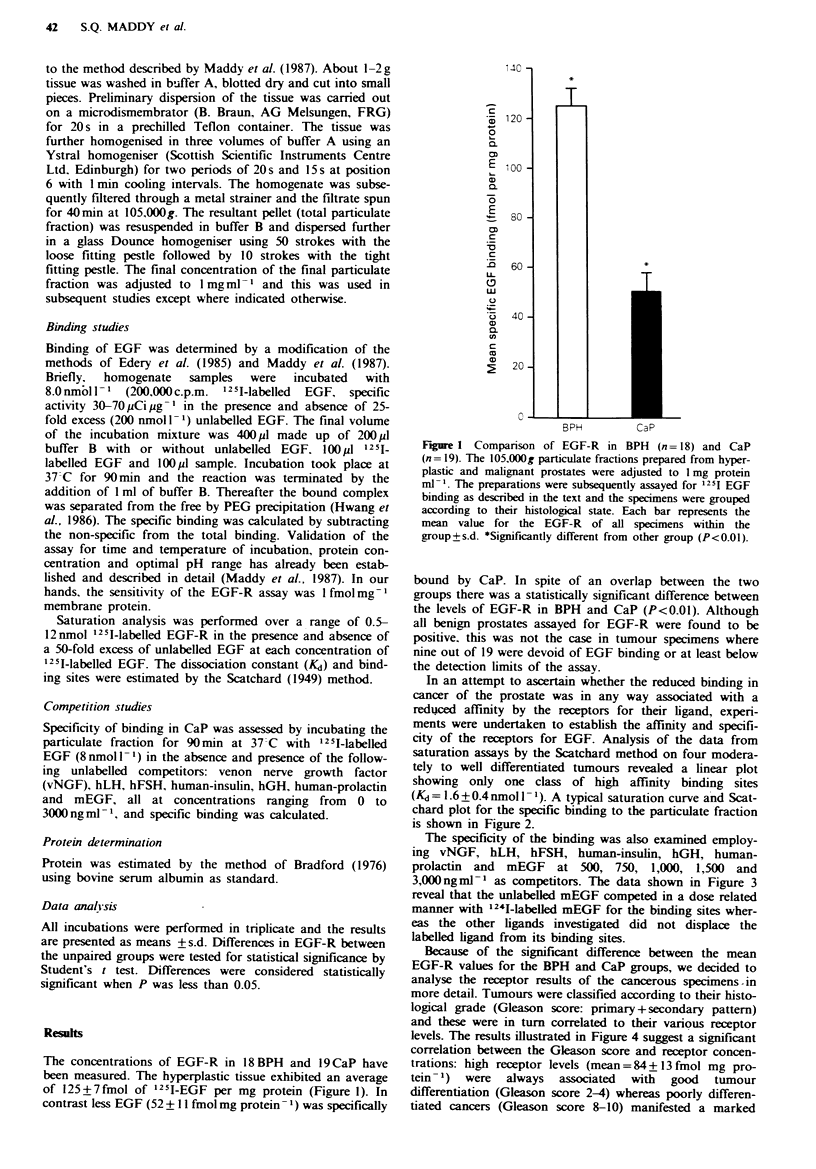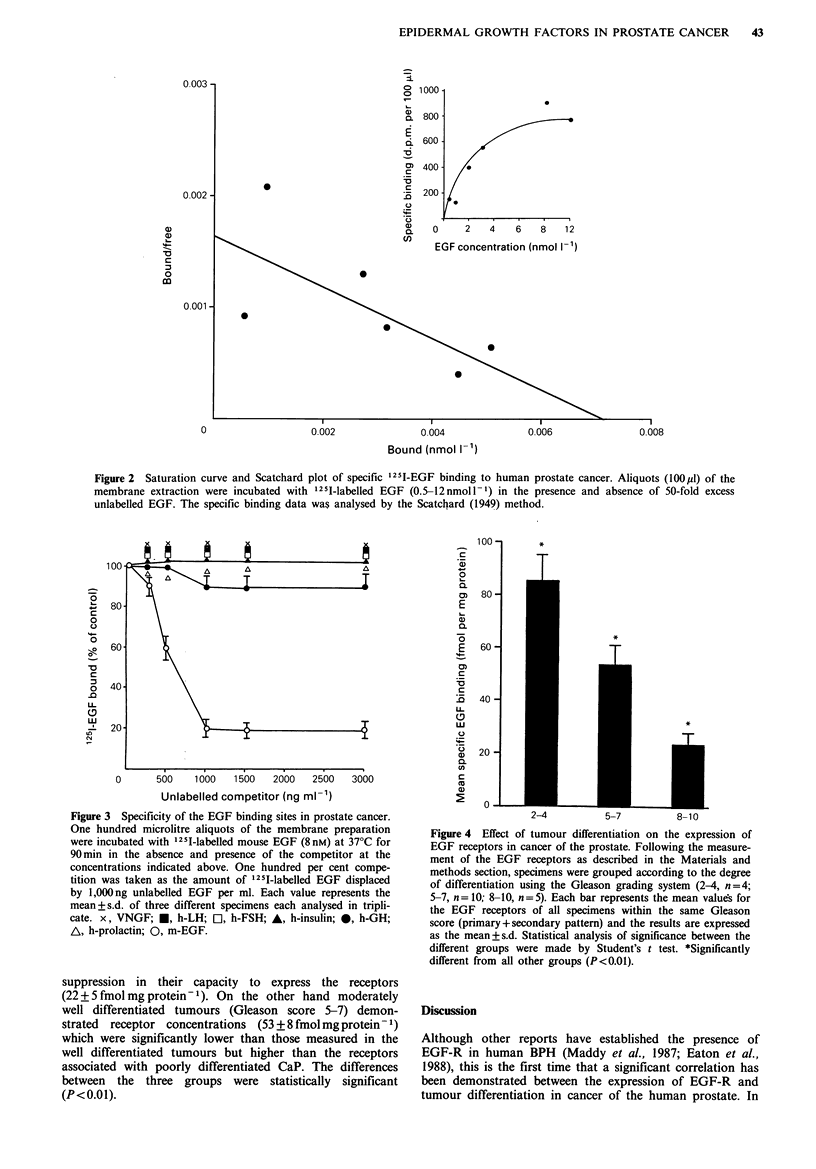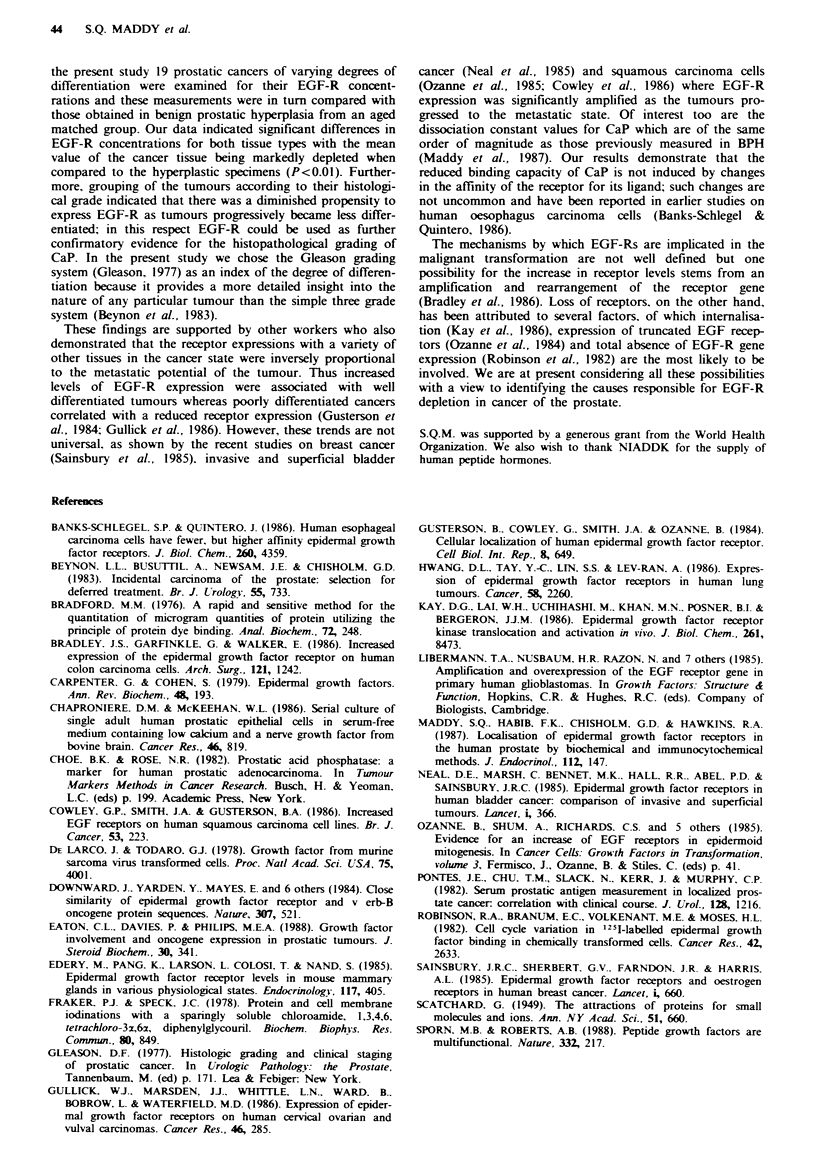# Epidermal growth factor receptors in human prostate cancer: correlation with histological differentiation of the tumour.

**DOI:** 10.1038/bjc.1989.216

**Published:** 1989-07

**Authors:** S. Q. Maddy, G. D. Chisholm, A. Busuttil, F. K. Habib

**Affiliations:** University Department of Surgery, Western General Hospital, Edinburgh, UK.

## Abstract

The presence of specific and high affinity epidermal growth factor receptors (EGF-R) has been demonstrated in human prostate cancer (CaP). Scatchard analysis of the binding data revealed a linear plot consistent with a single class of binding sites with a mean dissociation constant (Kd) +/- s.d. = 1.6 +/- 0.4 nmol 1-1. Additionally the binding was specific for EGF since no other competitor than EGF was able to displace the binding of the labelled ligand from its receptor. Comparison of the concentrations of EGF-R in tissues from 19 patients with CaP with those measured in a group of 18 patients with benign prostatic hyperplasia (BPH) reveal that the expression of EGF-R was significantly higher in BPH (mean +/- s.d. = 125 +/- 7 fmol mg protein-1) than in CaP (52 +/- 11 fmol mg protein-1; P less than 0.01). Furthermore, in CaP the expression of EGF-R varied according to the histological grade of the cancer: well differentiated tumours demonstrated more receptors (84 +/- 13 fmol mg protein-1) than poorly differentiated tumours (22 +/- 5 fmol mg protein-1; P less than 0.01). Clearly the depletion in the expression of EGF receptors in CaP is a function of the histological grade of the cancer and as such EGF receptors could be used as a biochemical marker for tumour differentiation.


Br. J. Cancer (1989), 60, 41–44 © The Macmillan Press Ltd., 1989

### Epidermal growth factor receptors in human prostate cancer: correlation with histological differentiation of the tumour

S.Q. Maddy[1], G.D. Chisholm[1], A. Busuttil[2] & F.K. Habib[1]

[1]*University Department of Surgery (WGH), Western General Hospital, Crewe Road, Edinburgh EH4 2XU, UK; and* [2]*Department of Forensic Medicine, University of Edinburgh, Medical School, Teviot Place, Edinburgh EH8 9AG, UK.*

**Summary** The presence of specific and high affinity epidermal growth factor receptors (EGF-R) has been demonstrated in human prostate cancer (CaP). Scatchard analysis of the binding data revealed a linear plot consistent with a single class of binding sites with a mean dissociation constant ($K_d$)$\pm$s.d.$=1.6\pm0.4$ nmol l$^{-1}$. Additionally the binding was specific for EGF since no other competitor than EGF was able to displace the binding of the labelled ligand from its receptor. Comparison of the concentrations of EGF-R in tissues from 19 patients with CaP with those measured in a group of 18 patients with benign prostatic hyperplasia (BPH) reveal that the expression of EGF-R was significantly higher in BPH (mean$\pm$s.d.$=125\pm7$ fmol mg protein$^{-1}$) than in CaP ($52\pm11$ fmol mg protein$^{-1}$; $P<0.01$). Furthermore, in CaP the expression of EGF-R varied according to the histological grade of the cancer: well differentiated tumours demonstrated more receptors ($84\pm13$ fmol mg protein$^{-1}$) than poorly differentiated tumours ($22\pm5$ fmol mg protein$^{-1}$; $P<0.01$). Clearly the depletion in the expression of EGF receptors in CaP is a function of the histological grade of the cancer and as such EGF receptors could be used as a biochemical marker for tumour differentiation.

The high incidence of prostatic cancer (CaP) has led to intensified study of this gland during the past two decades. The nature of the molecular changes responsible for the disease has attracted the attention of many biochemists and molecular biologists, but the complete solution to the cancer problem still eludes the best efforts of both scientists and clinicians. Early diagnosis and treatment are crucial. In this connection a considerable amount of work on a variety of substances synthesised and secreted by the prostate has been carried out in order to gain insight into the pathogenesis of this condition (Choe & Rose, 1982; Pontes *et al.*, 1982). Recently interest has focused on the many peptide growth factors because of their regulatory action on tissue growth (Sporn & Roberts, 1988).

Of particular interest is the epidermal growth factor (EGF) polypeptide (molecular weight 6,045 daltons) which stimulates proliferation and differentiation of a great variety of cell types (Carpenter & Cohen, 1979). The activities of EGF are mediated through its specific receptor, which is a 170 kilodalton glycoprotein located on the cell surface membrane. Several reports showed that the binding activity of EGF to the receptor in cancerous tissues was either decreased (De Larco & Todaro, 1978) or increased (Libermann *et al.*, 1985; Cowley *et al.*, 1986). Furthermore, the receptor expression has been correlated with the metastatic potential of the cancer (Gusterson *et al.*, 1984; Sainsbury *et al.*, 1985; Neal *et al.*, 1985; Cowley *et al.*, 1986; Gullick *et al.*, 1986). Recent evidence indicates that the oncogene v-erbB encodes a truncated EGF receptor which activates the tyrosine kinase to a 'turned on' state, thereby obviating the need for EGF stimulation (Downward *et al.*, 1984). The receptor was therefore thought to have some role in the biological behaviour of carcinoma cells. Although the localisation of EGF receptors (EGF-R) in human benign prostatic hyperplasia (BPH) has been established (Maddy *et al.*, 1987) and the importance of EGF in maintaining prostate cell proliferation has been demonstrated (Chaproniere & McKeehan, 1986) no reports have so far been published concerning the presence of EGF-R in human prostate cancer. In the present study we report for the first time the biochemical localisation of EGF-R in CaP and compare it with BPH.

Correspondence: F.K. Habib.
Received 7 December 1988, and accepted in revised form 13 February 1989.

### Materials and methods

### *Growth factor*

Mouse epidermal growth factor (mEGF) was purchased from Sigma, Poole, Dorset as electrophoretically pure and was iodinated without further processing. The iodination was carried out by the Iodogen method as described by Fraker & Speck (1978) and Maddy *et al.* (1987). The final specific activity of $^{125}$I-EGF varied between 30 and 70 $\mu$Ci $\mu$g$^{-1}$.

### *Other chemicals*

The following buffers were prepared and used throughout the investigation: 1. Buffer A containing tris (10 mmol l$^{-1}$), EDTA (1 mmol l$^{-1}$), EGTA (1 mmol l$^{-1}$), sucrose (0.25 mol l$^{-1}$) and phenylmethylsulphonyl fluoride (0.25 mmol l$^{-1}$); pH 7.4. 2. Buffer B containing tris (10 mmol l$^{-1}$), sodium chloride (0.9%), BSA (0.1%); pH 7.4. 3. 20% polyethylene glycol (PEG 8000 MW, Sigma). Solution was prepared using buffer B. 4. 10% PEG solution was prepared by diluting 20% PEG solution 1:2 using tris 10 mmol l$^{-1}$) containing (0.9%) sodium chloride; pH 7.4.

### *Prostate tissue*

Prostatic tissues were removed by transurethral resection from 18 patients between 62 and 85 years of age with BPH and 19 patients between 57 and 81 years with CaP. Large uncharred resection chippings were selected for our biochemical studies, placed in ice saline, blotted dry and weighed. Several sections from each specimen were sent for histological examination and grading of the cancerous tissue by the Gleason system (Gleason, 1977). This system is based upon the degree of glandular differentiation and the growth pattern of the tumour in relation to the prostatic stroma. The Gleason grade consists of two numbers, each between 1 and 5, which designate the primary and secondary pattern according to the amount of each present in the specimen. The sum of the two numbers is called the Gleason score and it may range from 2 to 10. The remainder of the tissue was either used fresh or snap frozen in liquid nitrogen and stored at $-70^{\circ}$C until analysis. None of the cancer patients had received any therapy – endocrine or otherwise – before entry into this study.

### *Tissue preparation*

The following procedures were carried out at 4°C according to the method described by Maddy et al. (1987). About 1–2 g tissue was washed in buffer A, blotted dry and cut into small pieces. Preliminary dispersion of the tissue was carried out on a microdismembrator (B. Braun, AG Melsungen, FRG) for 20 s in a prechilled Teflon container. The tissue was further homogenised in three volumes of buffer A using an Ystral homogeniser (Scottish Scientific Instruments Centre Ltd, Edinburgh) for two periods of 20 s and 15 s at position 6 with 1 min cooling intervals. The homogenate was subsequently filtered through a metal strainer and the filtrate spun for 40 min at 105,000 g. The resultant pellet (total particulate fraction) was resuspended in buffer B and dispersed further in a glass Dounce homogeniser using 50 strokes with the loose fitting pestle followed by 10 strokes with the tight fitting pestle. The final concentration of the final particulate fraction was adjusted to 1 mg ml$^{-1}$ and this was used in subsequent studies except where indicated otherwise.

*Binding studies*

Binding of EGF was determined by a modification of the methods of Edery et al. (1985) and Maddy et al. (1987). Briefly, homogenate samples were incubated with 8.0 nmol l$^{-1}$ (200,000 c.p.m. $^{125}$I-labelled EGF, specific activity 30–70 $\mu$Ci $\mu$g$^{-1}$ in the presence and absence of 25-fold excess (200 nmol l$^{-1}$) unlabelled EGF. The final volume of the incubation mixture was 400 $\mu$l made up of 200 $\mu$l buffer B with or without unlabelled EGF, 100 $\mu$l $^{125}$I-labelled EGF and 100 $\mu$l sample. Incubation took place at 37°C for 90 min and the reaction was terminated by the addition of 1 ml of buffer B. Thereafter the bound complex was separated from the free by PEG precipitation (Hwang et al., 1986). The specific binding was calculated by subtracting the non-specific from the total binding. Validation of the assay for time and temperature of incubation, protein concentration and optimal pH range has already been established and described in detail (Maddy et al., 1987). In our hands, the sensitivity of the EGF-R assay was 1 fmol mg$^{-1}$ membrane protein.

Saturation analysis was performed over a range of 0.5–12 nmol $^{125}$I-labelled EGF-R in the presence and absence of a 50-fold excess of unlabelled EGF at each concentration of $^{125}$I-labelled EGF. The dissociation constant ($K_d$) and binding sites were estimated by the Scatchard (1949) method.

*Competition studies*

Specificity of binding in CaP was assessed by incubating the particulate fraction for 90 min at 37°C with $^{125}$I-labelled EGF (8 nmol l$^{-1}$) in the absence and presence of the following unlabelled competitors: venon nerve growth factor (vNGF), hLH, hFSH, human-insulin, hGH, human-prolactin and mEGF, all at concentrations ranging from 0 to 3000 ng ml$^{-1}$, and specific binding was calculated.

*Protein determination*

Protein was estimated by the method of Bradford (1976) using bovine serum albumin as standard.

*Data analysis*

All incubations were performed in triplicate and the results are presented as means ±s.d. Differences in EGF-R between the unpaired groups were tested for statistical significance by Student's *t* test. Differences were considered statistically significant when *P* was less than 0.05.

### Results

The concentrations of EGF-R in 18 BPH and 19 CaP have been measured. The hyperplastic tissue exhibited an average of 125±7 fmol of $^{125}$I-EGF per mg protein (Figure 1). In contrast less EGF (52±11 fmol mg protein$^{-1}$) was specifically

**Figure 1** Comparison of EGF-R in BPH ($n$ = 18) and CaP ($n$ = 19). The 105,000 g particulate fractions prepared from hyperplastic and malignant prostates were adjusted to 1 mg protein ml$^{-1}$. The preparations were subsequently assayed for $^{125}$I EGF binding as described in the text and the specimens were grouped according to their histological state. Each bar represents the mean value for the EGF-R of all specimens within the group±s.d. *Significantly different from other group ($P$<0.01).

bound by CaP. In spite of an overlap between the two groups there was a statistically significant difference between the levels of EGF-R in BPH and CaP ($P$<0.01). Although all benign prostates assayed for EGF-R were found to be positive, this was not the case in tumour specimens where nine out of 19 were devoid of EGF binding or at least below the detection limits of the assay.

In an attempt to ascertain whether the reduced binding in cancer of the prostate was in any way associated with a reduced affinity by the receptors for their ligand, experiments were undertaken to establish the affinity and specificity of the receptors for EGF. Analysis of the data from saturation assays by the Scatchard method on four moderately to well differentiated tumours revealed a linear plot showing only one class of high affinity binding sites ($K_d$ = 1.6±0.4 nmol l$^{-1}$). A typical saturation curve and Scatchard plot for the specific binding to the particulate fraction is shown in Figure 2.

The specificity of the binding was also examined employing vNGF, hLH, hFSH, human-insulin, hGH, human-prolactin and mEGF at 500, 750, 1,000, 1,500 and 3,000 ng ml$^{-1}$ as competitors. The data shown in Figure 3 reveal that the unlabelled mEGF competed in a dose related manner with $^{124}$I-labelled mEGF for the binding sites whereas the other ligands investigated did not displace the labelled ligand from its binding sites.

Because of the significant difference between the mean EGF-R values for the BPH and CaP groups, we decided to analyse the receptor results of the cancerous specimens in more detail. Tumours were classified according to their histological grade (Gleason score: primary + secondary pattern) and these were in turn correlated to their various receptor levels. The results illustrated in Figure 4 suggest a significant correlation between the Gleason score and receptor concentrations: high receptor levels (mean = 84±13 fmol mg protein$^{-1}$) were always associated with good tumour differentiation (Gleason score 2–4) whereas poorly differentiated cancers (Gleason score 8–10) manifested a marked

**Figure 2** Saturation curve and Scatchard plot of specific $^{125}$I-EGF binding to human prostate cancer. Aliquots (100 $\mu$l) of the membrane extraction were incubated with $^{125}$I-labelled EGF (0.5–12 nmol l$^{-1}$) in the presence and absence of 50-fold excess unlabelled EGF. The specific binding data was analysed by the Scatchard (1949) method.

**Figure 3** Specificity of the EGF binding sites in prostate cancer. One hundred microlitre aliquots of the membrane preparation were incubated with $^{125}$I-labelled mouse EGF (8 nM) at 37°C for 90 min in the absence and presence of the competitor at the concentrations indicated above. One hundred per cent competition was taken as the amount of $^{125}$I-labelled EGF displaced by 1,000 ng unlabelled EGF per ml. Each value represents the mean ± s.d. of three different specimens each analysed in triplicate. ×, VNGF; ■, h-LH; □, h-FSH; ▲, h-insulin; ●, h-GH; △, h-prolactin; ○, m-EGF.

**Figure 4** Effect of tumour differentiation on the expression of EGF receptors in cancer of the prostate. Following the measurement of the EGF receptors as described in the Materials and methods section, specimens were grouped according to the degree of differentiation using the Gleason grading system (2–4, $n=4$; 5–7, $n=10$; 8–10, $n=5$). Each bar represents the mean values for the EGF receptors of all specimens within the same Gleason score (primary + secondary pattern) and the results are expressed as the mean ± s.d. Statistical analysis of significance between the different groups were made by Student's $t$ test. *Significantly different from all other groups ($P<0.01$).

suppression in their capacity to express the receptors (22 ± 5 fmol mg protein$^{-1}$). On the other hand moderately well differentiated tumours (Gleason score 5–7) demonstrated receptor concentrations (53 ± 8 fmol mg protein$^{-1}$) which were significantly lower than those measured in the well differentiated tumours but higher than the receptors associated with poorly differentiated CaP. The differences between the three groups were statistically significant ($P<0.01$).

### Discussion

Although other reports have established the presence of EGF-R in human BPH (Maddy *et al.*, 1987; Eaton *et al.*, 1988), this is the first time that a significant correlation has been demonstrated between the expression of EGF-R and tumour differentiation in cancer of the human prostate. In the present study 19 prostatic cancers of varying degrees of differentiation were examined for their EGF-R concentrations and these measurements were in turn compared with those obtained in benign prostatic hyperplasia from an aged matched group. Our data indicated significant differences in EGF-R concentrations for both tissue types with the mean value of the cancer tissue being markedly depleted when compared to the hyperplastic specimens ($P<0.01$). Furthermore, grouping of the tumours according to their histological grade indicated that there was a diminished propensity to express EGF-R as tumours progressively became less differentiated; in this respect EGF-R could be used as further confirmatory evidence for the histopathological grading of CaP. In the present study we chose the Gleason grading system (Gleason, 1977) as an index of the degree of differentiation because it provides a more detailed insight into the nature of any particular tumour than the simple three grade system (Beynon *et al.*, 1983).

These findings are supported by other workers who also demonstrated that the receptor expressions with a variety of other tissues in the cancer state were inversely proportional to the metastatic potential of the tumour. Thus increased levels of EGF-R expression were associated with well differentiated tumours whereas poorly differentiated cancers correlated with a reduced receptor expression (Gusterson *et al.*, 1984; Gullick *et al.*, 1986). However, these trends are not universal, as shown by the recent studies on breast cancer (Sainsbury *et al.*, 1985), invasive and superficial bladder cancer (Neal *et al.*, 1985) and squamous carcinoma cells (Ozanne *et al.*, 1985; Cowley *et al.*, 1986) where EGF-R expression was significantly amplified as the tumours progressed to the metastatic state. Of interest too are the dissociation constant values for CaP which are of the same order of magnitude as those previously measured in BPH (Maddy *et al.*, 1987). Our results demonstrate that the reduced binding capacity of CaP is not induced by changes in the affinity of the receptor for its ligand; such changes are not uncommon and have been reported in earlier studies on human oesophagus carcinoma cells (Banks-Schlegel & Quintero, 1986).

The mechanisms by which EGF-Rs are implicated in the malignant transformation are not well defined but one possibility for the increase in receptor levels stems from an amplification and rearrangement of the receptor gene (Bradley *et al.*, 1986). Loss of receptors, on the other hand, has been attributed to several factors, of which internalisation (Kay *et al.*, 1986), expression of truncated EGF receptors (Ozanne *et al.*, 1984) and total absence of EGF-R gene expression (Robinson *et al.*, 1982) are the most likely to be involved. We are at present considering all these possibilities with a view to identifying the causes responsible for EGF-R depletion in cancer of the prostate.

S.Q.M. was supported by a generous grant from the World Health Organization. We also wish to thank NIADDK for the supply of human peptide hormones.

**References**

BANKS-SCHLEGEL, S.P. & QUINTERO, J. (1986). Human esophageal carcinoma cells have fewer, but higher affinity epidermal growth factor receptors. *J. Biol. Chem.*, **260**, 4359.

BEYNON, L.L., BUSUTTIL, A., NEWSAM, J.E. & CHISHOLM, G.D. (1983). Incidental carcinoma of the prostate: selection for deferred treatment. *Br. J. Urology*, **55**, 733.

BRADFORD, M.M. (1976). A rapid and sensitive method for the quantitation of microgram quantities of protein utilizing the principle of protein dye binding. *Anal. Biochem.*, **72**, 248.

BRADLEY, J.S., GARFINKLE, G. & WALKER, E. (1986). Increased expression of the epidermal growth factor receptor on human colon carcinoma cells. *Arch. Surg.*, **121**, 1242.

CARPENTER, G. & COHEN, S. (1979). Epidermal growth factors. *Ann. Rev. Biochem.*, **48**, 193.

CHAPRONIERE, D.M. & McKEEHAN, W.L. (1986). Serial culture of single adult human prostatic epithelial cells in serum-free medium containing low calcium and a nerve growth factor from bovine brain. *Cancer Res.*, **46**, 819.

CHOE, B.K. & ROSE, N.R. (1982). Prostatic acid phosphatase: a marker for human prostatic adenocarcinoma. In *Tumour Markers Methods in Cancer Research*. Busch, H. & Yeoman, L.C. (eds) p. 199. Academic Press, New York.

COWLEY, G.P., SMITH, J.A. & GUSTERSON, B.A. (1986). Increased EGF receptors on human squamous carcinoma cell lines. *Br. J. Cancer*, **53**, 223.

DE LARCO, J. & TODARO, G.J. (1978). Growth factor from murine sarcoma virus transformed cells. *Proc. Natl Acad. Sci. USA*, **75**, 4001.

DOWNWARD, J., YARDEN, Y., MAYES, E. and 6 others (1984). Close similarity of epidermal growth factor receptor and v erb-B oncogene protein sequences. *Nature*, **307**, 521.

EATON, C.L., DAVIES, P. & PHILIPS, M.E.A. (1988). Growth factor involvement and oncogene expression in prostatic tumours. *J. Steroid Biochem.*, **30**, 341.

EDERY, M., PANG, K., LARSON, L. COLOSI, T. & NAND, S. (1985). Epidermal growth factor receptor levels in mouse mammary glands in various physiological states. *Endocrinology*, **117**, 405.

FRAKER, P.J. & SPECK, J.C. (1978). Protein and cell membrane iodinations with a sparingly soluble chloroamide, 1,3,4,6, *tetrachloro*-3α,6α, diphenylglycouril. *Biochem. Biophys. Res. Commun.*, **80**, 849.

GLEASON, D.F. (1977). Histologic grading and clinical staging of prostatic cancer. In *Urologic Pathology: the Prostate*, Tannenbaum, M. (ed) p. 171. Lea & Febiger: New York.

GULLICK, W.J., MARSDEN, J.J., WHITTLE, L.N., WARD, B., BOBROW, L. & WATERFIELD, M.D. (1986). Expression of epidermal growth factor receptors on human cervical ovarian and vulval carcinomas. *Cancer Res.*, **46**, 285.

GUSTERSON, B., COWLEY, G., SMITH, J.A. & OZANNE, B. (1984). Cellular localization of human epidermal growth factor receptor. *Cell Biol. Int. Rep.*, **8**, 649.

HWANG, D.L., TAY, Y.-C., LIN, S.S. & LEV-RAN, A. (1986). Expression of epidermal growth factor receptors in human lung tumours. *Cancer*, **58**, 2260.

KAY, D.G., LAI, W.H., UCHIHASHI, M., KHAN, M.N., POSNER, B.I. & BERGERON, J.J.M. (1986). Epidermal growth factor receptor kinase translocation and activation *in vivo*. *J. Biol. Chem.*, **261**, 8473.

LIBERMANN, T.A., NUSBAUM, H.R. RAZON, N. and 7 others (1985). Amplification and overexpression of the EGF receptor gene in primary human glioblastomas. In *Growth Factors: Structure & Function*, Hopkins, C.R. & Hughes, R.C. (eds). Company of Biologists, Cambridge.

MADDY, S.Q., HABIB, F.K., CHISHOLM, G.D. & HAWKINS, R.A. (1987). Localisation of epidermal growth factor receptors in the human prostate by biochemical and immunocytochemical methods. *J. Endocrinol.*, **112**, 147.

NEAL, D.E., MARSH, C. BENNET, M.K., HALL, R.R., ABEL, P.D. & SAINSBURY, J.R.C. (1985). Epidermal growth factor receptors in human bladder cancer: comparison of invasive and superficial tumours. *Lancet*, **i**, 366.

OZANNE, B., SHUM, A., RICHARDS, C.S. and 5 others (1985). Evidence for an increase of EGF receptors in epidermoid mitogenesis. In *Cancer Cells: Growth Factors in Transformation, volume 3*, Fermisco, J., Ozanne, B. & Stiles, C. (eds) p. 41.

PONTES, J.E., CHU, T.M., SLACK, N., KERR, J. & MURPHY, C.P. (1982). Serum prostatic antigen measurement in localized prostate cancer: correlation with clinical course. *J. Urol.*, **128**, 1216.

ROBINSON, R.A., BRANUM, E.C., VOLKENANT, M.E. & MOSES, H.L. (1982). Cell cycle variation in $^{125}$I-labelled epidermal growth factor binding in chemically transformed cells. *Cancer Res.*, **42**, 2633.

SAINSBURY, J.R.C., SHERBERT, G.V., FARNDON, J.R. & HARRIS, A.L. (1985). Epidermal growth factor receptors and oestrogen receptors in human breast cancer. *Lancet*, **i**, 660.

SCATCHARD, G. (1949). The attractions of proteins for small molecules and ions. *Ann. NY Acad. Sci.*, **51**, 660.

SPORN, M.B. & ROBERTS, A.B. (1988). Peptide growth factors are multifunctional. *Nature*, **332**, 217.